# Magnitude of Biofilm Formation and Antimicrobial Resistance Pattern of Bacteria Isolated from Urinary Catheterized Inpatients of Jimma University Medical Center, Southwest Ethiopia

**DOI:** 10.1155/2019/5729568

**Published:** 2019-02-10

**Authors:** Netsanet Awoke, Tesfaye Kassa, Lule Teshager

**Affiliations:** ^1^Department of Medical Laboratory Science, Dilla University, Dilla, Ethiopia; ^2^School of Medical Laboratory Science, Jimma University, P.O. Box 378, Jimma, Ethiopia; ^3^School of Medical Laboratory Science, Jimma University, P.O. Box 788, Jimma, Ethiopia

## Abstract

Biofilm formation is one of the features of most bacteria. Catheterization in medicine is a source of highly resistant bacterial infections, and those bacteria respond poorly to antimicrobial therapy. Bacterial biofilm features were not described from catheterized inpatients in Ethiopia as its formation is known to afford antimicrobial resistance and challenge patient management. The aim of this study was to isolate catheter-associated urinary bacterial pathogens, their biofilm formation, and antimicrobial susceptibility pattern among inpatients of Jimma University Medical Center (JUMC) in Southwest Ethiopia. A prospective cross-sectional study was conducted among urinary catheterized inpatients of JUMC from February to August 2016. A total of 143 study participants were enrolled consecutively in this study. Urine samples were collected from catheterized patients and processed using a standard bacteriological protocol for isolation and identification. Evaluation of *in vitro* biofilm formation and antimicrobial susceptibility pattern of uropathogenic bacteria was done using microtiter plates and disk diffusion method, respectively. Data were cleaned, coded, and entered into SPSS version 20 for analysis. All statistical test values of *p* < 0.05 were considered statistically significant. From all study participants, mean age was 44 years. Sixty bacterial strains were recovered from 57 urinary catheterized inpatients among which 54 of them were monomicrobial (94.7%). The remaining six bacterial strains were recovered from three study participants each with two bacterial isolates. The predominant bacterial isolates were Gram-negative bacteria with *E. coli* turning out first. About 80% of bacterial isolates were biofilm formers. The majority of the bacteria were resistant to commonly prescribed antimicrobial agents. In conclusion, the majority of bacterial uropathogen isolates were Gram-negative, biofilm formers, and resistant to commonly prescribed antimicrobial agents. Relatively ciprofloxacin, nitrofurantoin, and amikacin were highly effective against most isolated bacteria.

## 1. Introduction

Urinary tract infections (UTIs) account up to 40% of all hospital acquired infections around the globe, and more than 80% of nosocomial UTIs are usually associated with catheterization [1–3]. High prevalence of catheterization in a hospital setup leads to a large cumulative burden of catheter-associated UTIs with the resultant rise in morbidity and mortality [4]. The common uropathogenic bacteria known are *Escherichia coli*, *Klebsiella pneumoniae, Pseudomonas aeruginosa, Proteus* spp., and some other Gram-negative and Gram-positive bacteria [5, 6]. On the contrary, the emergence and spread of antimicrobial resistance among members of the family *Enterobacteriaceae*, particularly *E. coli* and *Klebsiella species*, are very common [7–10]. The high magnitude of antimicrobial resistance among catheter-associated UTIs has important economic implications particularly in developing countries where resources are lacking [3, 10].

Bacterial biofilm is a complex community of microorganisms with production of extracellular polysaccharide matrix on damaged tissue and surface of indwelling medical devices including urinary catheter. Despite catheter is generally critical indwelling medical device and indispensable in medicine, its prolonged use in hospitalized patients expose them to infection. This process allows bacteria to enter to the bladder and form biofilm either through migrating along the intra- or extraluminal parts of the catheter surface [11, 12]. This bacterial feature of biofilm production is very important in contributing to catheter-associated UTIs in hospital setting.

Bacterial biofilm development, on the other hand, is highly associated with the bacteria to afford inherent antimicrobial resistance such as the host's defense mechanisms and exogenous antimicrobial agents. This resistance makes the bacteria to be a major challenge for patient recovery [11]. It has been determined that biofilm-forming bacteria have shown resistance to antimicrobials as much as 1000 times more than their planktonic counterparts [1, 13]. Chronic and complicated UTIs can result in discomfort to the patient and prolonged hospital stay. This in turn ultimately increases hospital burdens, health care costs for diagnosis and treatment as well as higher morbidity and mortality of patients [1, 14–16].

The magnitude of catheter-associated UTIs problems remains underestimated largely in developing countries, whereas it is a persistently common problem [17, 18]. This is because of inadequate surveillance and absence of regular reporting system to national level. Its diagnosis and surveillance activities to guide preventions and interventions require expertise, facilities, and other resources [19]. On the other hand, detection of biofilm development of microorganisms in medicine has been progressing very slowly. This may be for the fact that overall knowledge about microorganisms living in biofilm communities is less among health professionals. This may be the perception that bacteria are single-celled organisms living an individual lifestyle [20]. For this reason, biofilm-related researches are important to bring front for the health professional attentiveness and future study initiation in Ethiopia.

Although community-acquired UTIs have been investigated in different groups of patients in Ethiopia, only very few studies have investigated and documented on the etiological agents of catheter-associated UTIs and their susceptibility pattern to antimicrobial agents [21, 22]. The updated pattern of antimicrobial susceptibility report helps in proper patient management as there were reports of variability in time and space. Moreover, there was no study on biofilm profiles of uropathogenic bacteria from catheterized patients in this study location in Jimma. Therefore, the aim of this study was to assess the distribution of urinary bacterial pathogens, their associated risk factors for acquisition of infection, biofilm formation, and drug susceptibility patterns to commonly used antimicrobial agents among urinary catheterized inpatients of JUMC, Southwest Ethiopia.

## 2. Materials and Methods

A prospective cross-sectional study was conducted in gynecology/obstetrics, surgical, and medical wards between February and August 2016, among urinary catheterized inpatients in JUMC, Southwest Ethiopia. The university center is a teaching institution and referral for 15 million populations with 650 beds in Southwest Ethiopia and provides specialized health services having surgical, medical, gynecological, maternity, pediatric, and other clinical and diagnostic departments. The hospital is providing services for approximately 15,000 inpatients, 160,000 outpatient attendants, 11,000 emergency cases, and 4500 deliveries per year from the catchment of about 15 million population (general background information on Jimma University Specialized Hospital, later named JUMC 2014: http://www.ju.edu.et/jimma-university-specialized-hospital-JUSH, accessed date: Oct 10, 2015).

### 2.1. Patient Recruitment

A total of 143 urinary catheterized inpatients were consecutively identified who was willing to participate in the study. Sociodemographic and clinical data were collected from the patient records and attending physicians using predesigned semistructured questionnaire adapted through reviewing published literatures and surveillance protocols [23, 24]. Initially, patients to be catheterized were screened for UTI by testing urine before and after urinary catheterization for clinical reasons. UTI-negative patients and who stayed on catheter for at least 48 hrs were included from the wards during the study period. Urine sample was collected prior to catheter removal from distal edge of the catheter tube with a sterile syringe after the sampling port of the catheter had been properly disinfected. Then, the urine was placed in a sterile, wide mouthed, dry, leak-proof plastic container from each study participants. Finally, the urine specimens were transported to medical microbiology laboratory of Jimma University immediately and analyzed within 2 hours.

### 2.2. Culture and Identification Procedures

Urine was inoculated directly onto blood agar, MacConkey agar, and Mannitol salt agar (media from Oxoid Ltd. Co, Basingstoke, Hampshire, England) using a calibrated inoculating loop capable to transfer 0.001 ml. Inoculated culture media were incubated in aerobic atmosphere at 37°C for 24 to 48 hours [25]. Identification of bacterial isolates were made based on their characteristic appearance on the respective media, Gram-staining, and biochemical reactions including catalase, coagulase, oxidase, indole production, citrate utilization, H_2_S production, motility, and other tests [25].

### 2.3. Antimicrobial Susceptibility Testing

Antimicrobial susceptibility was performed by the Kirby–Bauer disc diffusion technique following standard procedures on Muller–Hinton agar (Oxoid Ltd. Co, Hampshire, England) for only monomicrobial isolates, and zone of inhibition was compared with the standard value according to the criteria set by Clinical and Laboratory Standards Institute (CLSI) [26]. Multiple-drug resistance (MDR) was defined as bacterial resistance to three or more antimicrobial agents in different categories [7, 8, 26].

### 2.4. Quantitative Biofilm Formation Testing

All *in vitro* bacterial biofilm formations were studied only for those monomicrobial isolates. The ability of individual bacterial strain to form biofilm was evaluated by cultivating the bacteria on a 96-well clear flat-bottom polystyrene plastic microtiter plate (Tarsons, London, United Kingdom) as described elsewhere [27–29]. Briefly, standardized bacterial suspension in sterile normal saline adjusted to a 0.5 McFarland turbidity standard was prepared. Tryptic soya broth supplemented with 1% glucose was used to prepare bacterial suspension in 1 : 100 dilutions to add a final volume of 200 *µ*l per each well. This experiment was performed for each isolate in triplicates and incubated at 37°C for 24 h. The optical density (OD) was measured by using automated ELISA Autoreader (model 340, SpectroMax®) at a wavelength of 570 nm. The cutoff optical density (ODc) was calculated and defined as three standard deviations above the mean OD of the negative control. Isolates was classified as follows: bacterial OD < ODc = biofilm nonformer; OD > ODc, but <2 ODc = weak biofilm former; OD > 2 ODc but <4 ODc = moderate biofilm former and >4 ODc = strong biofilm former as described by Stepanović and his colleagues [29].

### 2.5. Quality Control

Standard reference strains including *S. aureus* (ATCC-25923), *E. coli* (ATCC-25922), and *P. aeruginosa* (ATCC-27853) were obtained from Ethiopia Public Health Institute laboratory and used as a quality control throughout the study for culture and antimicrobial susceptibility testing [16]. Moreover, control strains of *S. epidermidis* ATCC 12228 (biofilm nonformer or negative control) and *E. faecalis* ATCC 29212 (biofilm former or positive control) were used for *in vitro* biofilm evaluation procedures [28, 29].

### 2.6. Statistical Analysis

Data were coded and entered into SPSS version 20 for analysis. The chi-squared test, odds ratio with 95% CI, was used to screen possible associated factors among different variables. All statistical test values of *p* < 0.05 were considered statistically significant.

## 3. Results

### 3.1. Distribution of Bacterial Uropathogen

A total of 60 bacterial isolates were recovered from 57 (39.8%) study participants, and monomicrobial isolates were recovered from 54 (94.7%) participants. Six isolates were recovered from three catheterized inpatients each with the following isolates as a mixed infection: *S. aureus* and *Enterobacter* spp.; *E. coli* and *Klebssiella* spp.; and *Klebssiella* spp. and *S. aureus*. Of the total 60 bacterial isolates, 46 (76.7%) were Gram-negative with *E. coli* (19/60, 31.7%) being the predominant isolate (Table 1).

### 3.2. Distribution of Catheter-Associated Bacteriuria with Respect to Clinical Profiles

Among illnesses diagnosed during admission, patients with urogenital abnormality had highest catheter-associated bacteriuria (50.6%) with chi-square value (*χ* ^2^) = 11.57 and *p*=0.033. On the other hand, the higher prevalence of catheter-associated bacteriuria (45.3%) was observed among patients having underlining illness than study participants without this (36.7%); however, the observed difference was not significant, (*χ* ^2^) = 1.03, *p*=0.310. On the basis of study participants who had received antimicrobials, 27 (35.1%) of them have significant bacteriuria. In diabetic patients, catheter-associated bacteriuria (61.5%) was observed to be higher than those without the illness (37.7%) (Table 2). Patients on catheterization for about seven or more days had greater chance to develop catheter-associated bacteriuria (74.3%) compared with those with catheterization for less than four days, *χ* ^2^ = 24.846 at *p*=0.000. With respect to patients' hospital stay, study participants who had been hospitalized for 10 or more days developed higher catheter-associated bacteriuria (62.3%) than those with less than ten days stay, *p* < 0.001 (Table 2).

### 3.3. Pattern of Biofilm-Forming Uropathogenic Bacteria

From all bacterial isolates among urinary catheterized patients, forty-three (79.7%) of them were biofilm formers. From among Gram-negative and Gram-positive bacterial isolates, 34 (81%) and 9 (75%) of them were biofilm formers, respectively, with no significance difference (*p*=0.6516) (Table 3).

All clinical profiles of the patients including primary diagnosis upon admission, the presence of underlying illnesses, diabetes status, patients who received antimicrobial drugs, medical reason for catheterization, duration of catheterization, and length of hospital stay were not associated with biofilm formation patterns of bacterial isolates in this study (*p* > 0.15).

### 3.4. Antimicrobial-Resistance Profile of Bacterial Isolates

All bacterial isolates were 100% resistant to ampicillin, amoxicillin, and cephalexin. Gram-positive bacterial isolates have shown increased resistance to amoxicillin clavulanic acid (66.7%), tetracycline (67%), and trimethoprim/sulfamethoxazole (SXT) (75%). Furthermore, resistance of these Gram-positive isolates was seen to erythromycin, oxacillin, and gentamicin, each accounting for 92% and 100% resistance to penicillin (Table 4). Similarly, Gram-negative isolates had higher resistance to amoxicillin clavulanic acid (66.7%), SXT (76%), gentamicin (80.1%), and tetracycline (81.0%) (Table 5).

When all levels of biofilm-forming (strong, moderate, and weak) uropathogenic bacteria were brought together, those formers had higher antimicrobial resistance compared with nonbiofilm formers to amoxicillin-clavulanic acid (74.4% vs. 36.4%), ciprofloxacin (46.5% vs. 9.1%), ceftriaxone, and gentamicin with a *p* value of less than 0.05. However, in the remaining antimicrobial agents tested including nitrofurantoin, there was no significant difference in their action among biofilm and nonbiofilm former bacteria as shown in Table 6.

Multidrug-resistant features to different categories of antimicrobials were found among twenty-seven (62.8%) of biofilm-forming bacterial isolates with the majority of the drugs being commonly prescribed antimicrobials in this study area (Table 7).

## 4. Discussion

Urinary catheterization is one of the routine procedures used among patients through flexible tubes in healthcare facilities. As a result, catheter-associated UTI is becoming one of the major hospital-acquired infections reported globally. Its increased use in various cases and longer duration of catheterization allows the bacteria to colonize and adhere the urinary tract system [1, 15]. The bacteria in the process can infect the system to increase in prolonging hospital length of stay and ultimately increases burden and health care costs [14, 15]. Therefore, the present study was conducted to assess the distribution of catheter-associated bacterial pathogens, their biofilm formation capability, and antimicrobial susceptibility patterns among urinary catheterized inpatients of JUMC.

The spectrum of bacterial pathogens causing UTI may vary with time, the patient population, and study area. However, in most of the cases, Gram-negative bacteria were reported as common bacterial isolates [15, 30]. Similarly, in this study, Gram-negative bacteria were found to be the dominant etiologic agents (76.7%) of all bacterial isolates. Other researchers have also reported similar results [15, 21–23, 31–33].

In this study, the common uropathogenic bacterial species isolated were *E. coli* (31.6%) followed by *Klebsiella* spp. (23.3%). Similar studies have been reported elsewhere from Egypt [5], Kenya [34], and Bosnia [33] and three studies in Ethiopia [21, 22, 32]. The predominance of these bacteria in the gut as normal flora may result in an infection of the urinary tract by contaminating the urethra and ascends into the bladder. Furthermore, these pathogens may be acquired by cross-contamination from near patients since there were averagely more than six patients per ward room. Moreover, hospital personnel and exposure to contaminated equipment or surfaces may risk catheter-associated infection [1, 12]. Contrary to *E. coli* finding as the leading etiology, other studies conducted in Sudan [35], Nigeria [36, 37], and in Ethiopia [21] reported that *P. aeruginosa* or *S. aureus* was the frequent bacterial isolate. This difference in distribution of bacterial isolates may be due to difference in study area, duration of catheterization, and sample size.

Despite there is a lack of standardization in the measurement of *in vitro* bacterial biofilm formation worldwide, the feature of bacteria adherent to form biofilm can be appreciated by cultivation of bacteria on a plastic polystyrene microtiter plate [27, 29]. In the current study, about 80% of all bacterial isolates were capable to form biofilm formation with each of Gram-negative and Gram-positive isolates to 81% and 75%, respectively. This finding is comparable with studies carried out in Iraq [6] and three studies in India [6, 38, 39] where 69–72% of bacterial isolates were biofilm formers. However, lower biofilm-forming bacteria than that in our finding were reported in Egypt with 59.1% [5].

In the current study, *P. aeruginosa* and *Proteus* spp. were 100% biofilm formers followed by *E. coli* (77.8%) and *S. aureus* (80%). Similar patterns of biofilm formation were reported in studies conducted in India [38, 39]. In contrast, a study carried out in Egypt reported that higher biofilm production was among CONS (57.1%) followed by *Pseudomonas* (50.0%), *Klebsiella* (44.4%), *S. aureus* (42.9%), and *E. coli* (31.6%) [5]. This difference in biofilm-formation patterns among bacterial isolates may be due to differences in strain types, number of bacterial isolates, sample sizes, geographic locations, and methodological variations to assess biofilm formation.

The problems of bacterial drug resistance were globally documented particularly in healthcare-associated infections, and it is becoming one of health-security concerns [3, 19, 40, 41]. In this study, nearly two-third bacterial isolates showed multiple-drug resistance. This finding is similar to previous reports that showed resistance to various categories of antimicrobial drugs including penicillin and tetracycline at various geographic locations elsewhere including in Ethiopia [21, 22, 32, 33, 37, 42]. The possible explanation for higher resistance of bacteria to those drugs may be widespread and indiscriminate use as well as its ease of accessibility over the counter in pharmacies, which can lead to a shift to increase in resistant microbes [19, 40, 41]. In this study, 54% of the patients were empirically treated with at least one of those commonly prescribed antimicrobials.

In this study, more than two-thirds of the isolates were resistant to SXT (76%), amoxicillin-clavulanic acid (66.7%), tetracycline (81.0%), and gentamicin (80.1%) among Gram-negative bacteria. Studies carried out in different locations reported resistance to SXT (87.3–100%), amoxacillin-clavulanic acid (86%), gentamicin (81–91%), and tetracycline (89.1–100%) [22, 30, 32, 33, 37, 42]. Moreover, the Gram-negative bacteria isolated in this study were resistance to ceftriaxone (54.7%) and ciprofloxacin (40.4%). This finding is comparable to reports from the previous studies conducted in Ethiopia [22]. However, higher resistance to cephalosporin and fluoroquinolones was reported from various studies in the range between 56–100% and 66.7–81.1%, respectively [30, 37, 42–44]. This increase in resistance might result from poorly guided and frequent use of antimicrobial prophylaxis and empiric therapy with cephalosporin and fluoroquinolones in the last few years contributing to this likely rise in ceftriaxone and ciprofloxacin resistance [1, 12]. Besides, the present study indicated that nitrofurantoin and amikacin had relatively low resistance to Gram-negative bacteria about 38.1%, and 19.1%, respectively. Thus, nitrofurantoin and amikacin are good choice for empirical management of catheter-associated UTIs in our setting.

All Gram-positive bacteria in this study were 100% resistant to penicillin G, ampicillin, amoxicillin, and cephalexin and 92% resistant to erythromycin and oxacillin. Similar observations were reported in some previous studies in the same study setting in Ethiopia [22, 32], Nigeria [37], and India [30]. On the other hand, the majority of the Gram-positive isolates were resistant to gentamicin (92%), SXT (75%) and tetracycline and amoxicillin-clavulanic acid (each 67%). However, these bacteria were relatively less resistant to ciprofloxacin (33.3%) and ceftriaxone (50%). This may be in contrast with the previous report in Ethiopia that showed higher resistance to ciprofloxacin (100%) [32]. Hence, this research finding in our location may not guarantee future use of these drugs as the shift towards higher resistance may happen quickly.

In this study, catheterization for seven or more days was identified as a risk factor for development of bacteriuria among urinary catheterized inpatients of JUMC. Similar findings have been reported in various countries around the world [23, 31, 34, 45–51] including Ethiopia [32]. This may be because the longer time the catheter remains in the urinary system, it is highly likely bacteria can colonize, accumulates in the residual urine in the bladder, adhere or aggregate, and form complex communities of bacterial species called biofilms [11, 20].

In this study, the overall biofilm-forming bacterial isolates had higher antimicrobial resistance than that of nonbiofilm formers to amoxicillin-clavulanic acid (74.4% vs. 36.4%) and ciprofloxacin (46.5% vs. 9.1%), respectively. Similar studies carried out in different locations showed that biofilm formers have higher resistance features compared with those of nonbiofilm formers to amoxicillin clavulanic acid [19, 38, 52]. Moreover, multidrug resistance was observed among biofilm-forming bacterial isolates than their counter parts to amoxicillin and cephalexin. This higher antimicrobial resistance among biofilm former may emerge from increased properties of efflux mechanism. In addition, it may be also associated with higher plasmid transfer, modified target genes, and metabolic pathway that allow for resistance to antimicrobials [5, 11]. About two-third (62.8%) of the biofilm former isolates were multidrug resistant to at least three or more antimicrobials. This is an alarming record where selection of efficacious antimicrobial may need to be guided by culture-based methods and *in vitro* susceptibility studies.

## 5. Conclusions

This study revealed that the overall prevalence of catheter-associated bacteriuria was 39.8%, and the predominant bacterial isolates were Gram negative such as *E. coli* and *Klebsiella* spp. About 80% of bacterial isolates from catheter-associated UTI were biofilm formers. In addition, this study indicated that bacterial isolates had higher prevalence of resistance to commonly prescribed antimicrobial agents. Relatively, ciprofloxacin, nitrofurantoin, and amikacin seem to be useful against most isolated Gram-negative and Gram-positive bacteria and can be used as drug of choice for the empirical management of catheter-associated UTIs. Awareness is required to minimize occurrence of biofilm-forming uropathogens among urinary catheterized patients by health professionals.

## Figures and Tables

**Table 1:** Distribution of uropathogenic bacterial isolates among urinary catheterized inpatients of JUMC, Southwest Ethiopia, from February to August 2016.

Bacterial isolates (*N*=60)	Frequency (%)
*Gram-negative bacteria*	**46 (76.7)**
*E. coli*	19 (31.7)
*Klebsiella* spp.	14 (23.3)
*Proteus* spp.	4 (6.7)
*P. aeruginosa*	3 (5.0)
*Citrobacter* spp.	3 (5.0)
*Enterobacter* spp.	3 (5.0)
*Gram-positive bacteria*	**14 (23.3)**
CONS	7 (11.66)
*S. aureus*	7 (11.66)
Grand total	60 (100)

**Table 2:** Distribution of catheter-associated significant bacteriuria with respect to clinical profiles of inpatients at JUMC, Southwest Ethiopia, from February to August 2016.

Variables	Catheter-associated significant bacteriuria				
	Yes (%)	No (%)	Total	*χ* ^2^	*p* value
*Primary diagnosis upon admission*					
Urogenital abnormality	44 (50.6)	43 (49.4)	87	11.56	0.033
Leg or head injury	5 (21.7)	18 (78.3)	23		
Malignancy	5 (29.4)	12 (70.6)	17		
Appendicitis or bowel obstruction	1 (11.1)	8 (88.9)	9		
Chronic heart failure	2 (28.6)	5 (71.4)	7		
*Presence of underlying illness*					
Yes	24 (45.3)	29 (54.7)	53	1.03	0.310
No	33 (36.7)	57 (63.3)	90		
*Diabetes status*					
Diabetic	8 (61.5)	5 (38.5)	13	2.80	0.104
Nondiabetic	49 (37.7)	81 (62.5)	130		
*Antimicrobial drug received*					
Yes	27 (35.1)	50 (64.9)	77	1.60	0.207
No	30 (45.5)	36 (54.5)	66		
*Reason for catheterization*					
Pre- or postoperative drainage	25 (34.7)	47 (65.3)	72	1.71	0.636
Urine output measurement	4 (44.4)	5 (55.6)	9		
Incontinence	12 (42.9)	16 (57.1)	28		
Urinary retention	16 (47.1)	18 (52.9)	34		
*Duration of catheterization/day*					
<4 days	14 (23)	47 (77)	61	24.84	<0.001
4–6 days	17 (36.2)	30 (63.8)	47		
≥7 days	26 (74.3)	9 (25.7)	35		
*Length of hospital stay/day*					
<10 days	24 (26.7)	66 (73.3)	90	17.63	<0.001
≥10 days	33 (62.3)	20 (37.7)	53		
*Total*	**57 (39.8)**	**86 (60.2)**	**143**		

**Table 3:** Biofilm formation patterns of bacterial isolates among urinary catheterized inpatients of JUMC, Southwest Ethiopia, from February to August 2016.

Bacterial isolates	Biofilm formation patterns					
	BF			NBF (%)	Chi-square test	
	SBF (%)	MBF (%)	WBF (%)		*χ* ^2^	*p* value
*E. coli* (*N*=18)	3 (16.7)	2 (11.1)	9 (50)	4 (22.2)	25.83	0.213
*Klebsiella* spp. (*N*=12)	5 (41.7)	1 (8.3)	3 (25)	3 (25)		
*P. aeruginosa* (*N*=3)	2 (66.7)	1 (33.3)	—	—		
*Proteus* spp. (*N*=4)	2 (50)	—	2 (50)	—		
*Citrobacter* spp. (*N*=3)	—	1 (33.3)	1 (33.3)	1 (33.3)		
*Enterobacter* spp. (*N*=2)	1 (50)	1 (50)	—	—		
CONS (*N*=7)	2 (28.6)	3 (42.9)	—	2 (28.6)		
*S. aureus* (*N*=5)	4 (80)	—	—	1 (20)		
*Grand total* (*N*=54)	19 (35.2)	9 (16.7)	15 (27.8)	11 (20.3)		

BF: biofilm formers; NBF: nonbiofilm formers; SBF: strong biofilm formers; MBF: moderate biofilm formers; WBF: weak biofilm formers; N: number; CONS: coagulase-negative staphylococci.

**Table 4:** Antimicrobial-resistance pattern of Gram-positive bacteria isolated from urinary catheterized inpatients of JUMC, Southwest Ethiopia, from February to August 2016.

Antimicrobials	Antimicrobial resistance (%)		
	*S. aureus* (*N*=5) (*N* (%))	CONS (*N*=7) (*N* (%))	Total (*N*=12) (*N* (%))
AMP	5 (100)	7 (100)	12 (100)
AML	5 (100)	7 (100)	12 (100)
AMC	3 (60)	5 (71)	8 (66.7)
CL	5 (100)	7 (100)	12 (100)
CIP	2 (40)	2 (28.6)	4 (33.3)
CRO	2 (40)	4 (57.1)	6 (50)
GN	5 (100)	6 (86)	11 (92)
F	1 (20)	2 (29)	3 (25)
SXT	5 (100)	4 (57)	9 (75)
Tet	3 (60)	5 (71)	8 (67)
AK	1 (20)	2 (29)	3 (25)
E	4 (80)	7 (100)	11 (92)
P	5 (100)	7 (100)	12 (100)
Ox	5 (100)	6 (86)	11 (92)

AMP: ampicillin; AML: amoxicillin; AMC: amoxicillin-clavulanic acid; CL: cephalexin; CIP: ciprofloxacin; GN: gentamicin; F: nitrofurantoin; SXT: trimethoprim/sulfamethoxazole; CRO: ceftriaxone; Tet: tetracycline; AK: amikacin; E: erythromycin; P: penicillin; Ox: oxacillin.

**Table 5:** Antimicrobial-resistance pattern of Gram-negative bacteria isolated from urinary catheterized inpatients of JUMC, Southwest Ethiopia, from February to August 2016.

Antimicrobials	Antimicrobial resistance (%) of gram-negative bacterial isolates						
	*E. coli* (*N*=18)	*Klebssiella* spp. (*N*=12)	*P. aeruginosa* (*N*=3)	*Proteus* spp. (*N*=4)	*Citrobacter* spp. (*N*=3)	*Enterobacter* spp. (*N*=2)	Total (*N*=42)
AMP	18 (100)	12 (100)	3 (100)	4 (100)	3 (100)	2 (100)	42 (100)
AML	18 (100)	12 (100)	3 (100)	4 (100)	3 (100)	2 (100)	42 (100)
AMC	11 (61.1)	8 (66.7)	2 (66.7)	3 (75)	2 (66.7)	2 (100)	28 (66.7)
CL	18 (100)	12 (100)	3 (100)	4 (100)	3 (100)	2 (100)	42 (100)
CIP	7 (38.9)	4 (33.3)	2 (66.7)	2 (50)	1 (33.3)	1 (50)	17 (40.4)
CRO	10 (55.6)	7 (58.3)	2 (66.7)	2 (50)	1 (33.3)	1 (50)	23 (54.7)
GN	14 (78)	9 (75)	3 (100)	4 (100)	3 (100)	1 (50)	34 (80.1)
F	6 (33.3)	5 (41.7)	1 (33.3)	2 (50)	1 (33.3)	1 (50)	16 (38.1)
SXT	15 (83)	9 (75)	2 (66.7)	3 (75)	2 (66.7)	1 (50)	32 (76.0)
Tet	15 (83)	8 (66.7)	3 (100)	4 (100)	2 (66.7)	2 (100)	34 (81.0)
AK	4 (22)	1 (8.3)	1 (25)	1 (25)	1 (33.3)	—	8 (19.1)

**Table 6:** Antimicrobial-resistance patterns between biofilm and nonbiofilm former bacterial isolates from urinary catheterized inpatients of JUMC, Southwest Ethiopia, 2016.

Antimicrobials	Susceptibility pattern^#^	Biofilm formation pattern and antimicrobial resistance (%)		Chi-square	
		BP (*N*=43)	NBP (*N*=11)	Odds ratio	*p* value
AMC	Sensitive	11 (25.6)	7 (63.6)	5.71	0.017^*∗*^
	Resistant	32 (74.4)	4 (36.4)		

CIP	Sensitive	23 (53.5)	10 (90.9)	5.16	0.023^*∗*^
	Resistant	20 (46.5)	1 (9.1)		

CRO	Sensitive	17 (39.5)	8 (72.7)	3.88	0.049^*∗*^
	Resistant	26 (60.5)	3 (27.3)		

GN	Sensitive	5 (11.6)	4 (38.4)	3.86	0.0495^*∗*^
	Resistant	38 (88.4)	7 (63.6)		

F	Sensitive	26 (60.5)	9 (81.8)	1.75	0.186^*∗*^
	Resistant	17 (39.5)	2 (18.2)		

SXT	Sensitive	9 (20.9)	4 (36.4)	1.14	0.285^*∗∗*^
	Resistant	34 (79.1)	7 (63.6)		

Tet	Sensitive	8 (18.6)	4 (36.4)	1.60	0.206^*∗∗*^
	Resistant	35 (81.4)	7 (63.6)		

AK	Sensitive	33 (76.7)	10 (90.9)	1.08	0.298^*∗∗*^
	Resistant	10 (23.3)	1 (9.1)		

^*∗*^Biofilm-forming bacteria with tendency to become more antimicrobial resistant compared with that of nonbiofilm producing ones (*p* ≤ 0.05). ^*∗∗*^Nonsignificant. ^#^Very little intermediate susceptibilities of isolate results were merged to susceptible category.

**Table 7:** Multiple-drug resistant patterns of biofilm-forming bacterial uropathogens isolated from inpatients of JUMC, Southwest Ethiopia, from February to August 2016.

Antimicrobials	Resistance (%) (*N*=43)
AMP, AML, CL	43 (100)
SXT, Tet	28 (65.1)
SXT, Tet, G	27 (62.8)
SXT, Tet, G, AMC	21 (48.8)
SXT, Tet, G, AMC, CRO	16 (37.2)
SXT, Tet, G, AMC, CRO, CIP	10 (23.3)
SXT, Tet, G, AMC, CRO, CIP, F	5 (11.6)
SXT, Tet, G, AMC, CRO, CIP, F, AK	1 (2.3)

## Data Availability

The raw data from inpatients used to support the findings of this study are available from the corresponding author upon request.

## Supplementary Materials

Supplementary Materials Supplementary Table 1: distribution of biofilm formation pattern bacteria with respect to clinical profiles of inpatients of JUMC, Southwest Ethiopia, February to August 2016.
