# Supplementary material for: Magnitude of Biofilm Formation and Antimicrobial Resistance Pattern of Bacteria Isolated from Urinary Catheterized Inpatients of Jimma University Medical Center, Southwest Ethiopia
Source: Int J Microbiol. 2019 Feb 10;2019:5729568. doi: 10.1155/2019/5729568 (PMC6387724; doi:10.1155/2019/5729568)
Supplement: Supplementary Materials — Supplementary Table 1: distribution of biofilm formation pattern bacteria with respect to clinical profiles of inpatients of JUMC, Southwest Ethiopia, February to August 2016. [file 5729568.f1.pdf]

Supplementary Table 1: Distribution of biofilm formation pattern bacteria with respect clinical profiles of inpatients of JUMC, southwest Ethiopia; February - August, 2016.

variables	Biofilm formation pattern					
	SBF (%)	MBF (%)	WBF (%)	NBF (%)	χ^2	P -value
**Primary diagnosis upon admission**						
Uro-genital abnormality	15(34.9)	7(16.3)	14(32.6)	7(16.3)	9.87	0.627
Leg or head injury	2(50)	1(25)	0(0)	1(25)		
Malignancy	1(25)	1(25)	0(0)	2(50)		
Appendicitis/bowel obstruction	0	0	1(100)	0		
Chronic heart failure	1(50)	0	0	1(50)		
**Presence of underlying illness**						
Yes	10(43.5)	3(13.0)	4(17.4)	6(26.1)	3.29	0.348
No	9(29)	6(19.4)	11(35.5)	5(16.1)		
**Diabetes status**						
Diabetic	3(37.5)	2(25.0)	1(12.5)	2(25)	1.94	0.731
Non diabetic	16(34.8)	7(15.2)	14(30.4)	9(19.6)		
**Antimicrobial drug received**						
Yes	13(48.1)	4(14.8)	6(22.2)	4(14.8)	4.10	0.250
No	6(22.2)	5(18.5)	9(33.3)	7(25.9)		
**Reason for catheterization**						
Pre or post-operative drainage	10(43.5)	4(17.4)	5(21.7)	4(17.4)	7.24	0.612
Urine output measurement	1(25)	0	2(50)	1(25)		
Incontinence	4(36.4)	1(9.1)	5(45.5)	1(9.1)		

Urinary retention	4(25)	4(25)	3(18.8)	5(31.2)		
**Duration of catheterization /day**						
<4 days	4(30.8)	2(15.4)	5(38.5)	2(15.4)		
4-6 days	9(52.9)	2(11.8)	3(17.6)	3(17.6)	4.46	0.614
≥ 7 days	6(25)	5(20.6)	7(29.2)	6(25)		
**Length of hospital stay/ day**						
<10 days	10(43.5)	1(4.3)	8(34.8)	4(17.4)	5.31	0.150
≥10 days	9(29.0)	8(25.8)	7(22.6)	7(22.6)		
**Total**	**19(35.2)**	**9(16.7)**	**15(27.8)**	**11(20.4)**		
